# The influence of psilocybin on subconscious and conscious emotional learning

**DOI:** 10.1016/j.isci.2024.110034

**Published:** 2024-05-19

**Authors:** Andrea F. Casanova, Andres Ort, John W. Smallridge, Katrin H. Preller, Erich Seifritz, Franz X. Vollenweider

**Affiliations:** 1 Neurophenomenology of Consciousness Lab, Department of Psychiatry, Psychotherapy and Psychosomatics, Psychiatric Hospital, University of Zurich, Zurich, Switzerland; 2 Department of Psychiatry, Psychotherapy and Psychosomatics, Psychiatric Hospital, University of Zurich, Zurich, Switzerland

**Keywords:** Pharmacology, Natural sciences, Biological sciences, neuroscience, clinical neuroscience

## Abstract

Serotonergic psychedelics hold promise as a treatment modality for various psychiatric disorders and are currently applied in psychedelic-assisted psychotherapy. We investigated the learning effects of the serotonin receptor agonist psilocybin in a probabilistic cue-reward task with emotional cues in the form of neutral or fearful faces, presented either consciously or subconsciously. This study represents the first investigation into reinforcement learning with psilocybin. Across different dosages, psilocybin preserved learning effects and was statistically noninferior compared to placebo, while suggesting a higher exploratory behavior. Notably, the 20 mg group exhibited significantly better learning rates against the placebo group. Psilocybin induced inferior results with subconscious cues compared to placebo, and better results with conscious neutral cues in some conditions. These findings suggest that modulating serotonin signaling in the brain with psilocybin sufficiently preservers reinforcement learning.

## Highlights

• With Psilocybin learning was preserved and statistically noninferior to placebo
• Decision-making with psilocybin suggests a higher exploratory behavior
• Higher dosage of psilocybin improved learning performance against placebo
• Emotional cues in a learning task affected psilocybin more than placebo

## Graphical abstract

**Figure:**
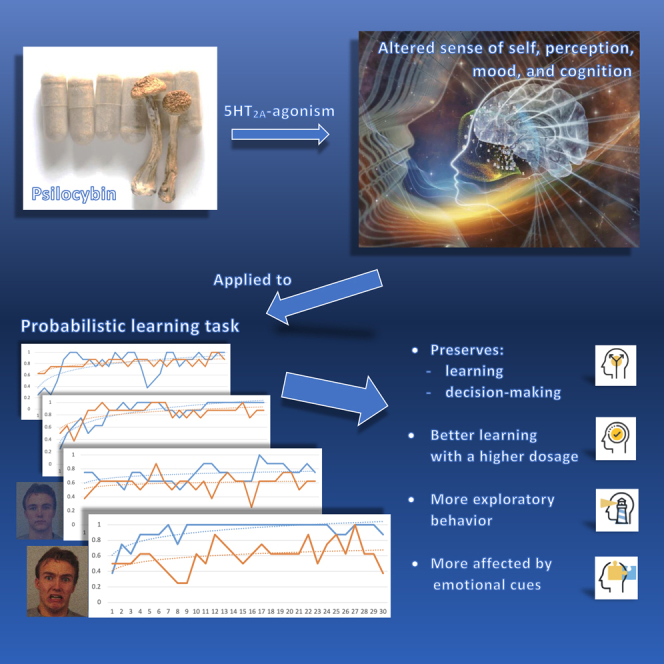

## Introduction

Psilocybin (*4-phosphoryloxy-dimethyltryptamine*) is a serotonergic tryptamine hallucinogen structurally related to serotonin (5-HT; *5-hydroxytryptamine*). It is found in "magic mushrooms" (e.g., *Psilocybe cubensis*) and has a rich history of ritual use in various cultures.^1^ When ingested, psilocybin is converted into its active form psilocin (*4-hydroxy-dimethyltryptamine*),^2^^,^^3^ which produces its mind-altering effects through various serotonin receptors and downstream effects on the GABAergic,^4^ dopaminergic,^5^ and glutamatergic systems.^4^ Healthy humans ingesting psilocybin can experience profound changes in the sense of self, perception, mood, and cognition, including alterations in self-referential processing.^6^^,^^7^

In recent years, psychedelics have reemerged as promising treatments for various psychiatric disorders.^8^ Psilocybin and lysergic acid diethylamide (LSD) have been shown to significantly reduce symptoms of depression and anxiety in patients with major depressive disorder (MDD),^9^^,^^10^^,^^11^^,^^12^ anxiety,^13^ and substance use disorder.^14^ Interestingly, psychedelics have been shown to enhance synaptic neuroplasticity in animals,^15^^,^^16^^,^^17^ which might drive cognitive adaptations while facilitating therapeutic action through effects on learning and offer an original mechanism for the lasting beneficial outcomes in patients suffering from depression. A further factor potentially contributing to therapeutic effects was that psilocybin decreases connectivity between brain regions involved in emotional processing, such as when recognizing angry faces.^14^^,^^16^ This shift in processing emotional information biases positive and neutral information over negative information, correlating with an increase in positive mood.^8^

The psychedelic compound LSD was found to increase both reward and punishment learning rate in a learning task.^18^ By promoting a state of heightened plasticity and increased exploratory behavior, LSD led to an enhanced rate of belief updating. These effects are thought to facilitate the revision of maladaptive associations.^18^ While psychedelics have been demonstrated to acutely alter cognitive processing at different levels, such as attention,^19^ working memory,^20^ and goal-directed^21^ and prosocial behavior,^22^^,^^23^ little is known about the effects of psilocybin on learning, particularly concerning the role of conscious versus subconscious emotional processing.

While LSD has a broader serotonin receptor binding profile, including activity at dopamine receptors, psilocybin primarily acts via partial agonism of 5-HT_2A_ receptors in cortical and subcortical brain regions.^24^^,^^25^ Furthermore, it exerts modulatory effects on the 5-HT_1A_-receptor^26^ and others, including the 5-HT_2B/2C/6/7_-receptors.^25^^,^^27^ Although having no direct affinity for dopamine receptors, psilocybin has been found to elevate striatal dopamine (DA) concentration,^5^ with the 5-HT_2A_ receptor hypothesized to activate the DA system.^28^ The mesocorticolimbic DA system plays a pivotal role in learning by capturing novelty through reward prediction errors (PE), measured by characteristic dopaminergic firing patterns. Increased DA firing is behavior-promoting whenever a reward is higher than expected (high striatal D_1_-receptor activation on direct pathway neurons leading to action promotion), while decreased firing rate is behavior-discouraging if a reward is less than expected (low striatal D_2_-receptor activation on indirect pathway neurons leading to action suppression).^29^ Serotonin is believed to maintain a delicate balance with DA in learning and behavior reinforcement. Studies indicate that depleting 5-HT levels can dysregulate the activity of DA neurons, leading to heightened responsiveness to immediate rewards and decreased inclination toward long-term goals. Conversely, increasing 5-HT levels with prolonged selective serotonin reuptake inhibitor (SSRI) administration can inhibit the activity of DA neurons, leading to a decreased emphasis on immediate rewards, promoting patience for future rewards, and amplifying motivation toward long-term goals.^30^

To test the effects of a substance on learning, reinforcement learning can be applied – a feedback-driven learning paradigm aimed at maximizing long-term rewards by learning optimal behavior through trial-and-error. Probabilistic learning tasks model behavioral dynamics in decision-making fraught with uncertainty, employing Bayesian inference to adapt priors continuously in uncertain environments, steering behavior toward desired outcomes. Agents simultaneously juggle uncertainty while associating stimuli with affective values, thereby enforcing associations between an unconditioned stimulus (a reward) and a conditioned stimulus (a cue). Emotional faces serve as cues in learning tasks, influencing the emotional valence of stimuli and the processing and retention of information.^31^ Emotional cues typically enhance learning, with negative cues (e.g., fearful faces) yielding additional improvement compared to neutral cues.^32^ However, the duration of the presentation of cues also plays a role. Some studies observed subconscious cues to impede learning performance compared to conscious cues, demanding more cognitive resources for processing.^33^^,^^34^ Dysfunctions in reinforcement learning have been implicated in a range of psychiatric disorders, including addiction and major depressive disorder (MDD),^35^ whereas prolonged administration of SSRIs in MDD was associated with reduced reinforcement learning by blunting emotional responses, thereby impairing learning from negative stimuli while showing negligible effects on learning with positive stimuli.^36^^,^^37^ As emotional blunting is not observed with psilocybin in contrast to SSRIs,^38^ we sought to compare the extent of valence-specific learning effects against placebo. In particular, in this study, we investigate the potential therapeutic implication of psilocybin on learning by comparing it to a placebo in a probabilistic cue-reward task with subconscious and conscious emotional cues.

We had three hypotheses. First, we hypothesized that by decreasing the salience of learning-enhancing fearful faces, psilocybin would impede learning with negative stimuli. Second, based on the observed neuroplastic effect in animals but not yet studied in humans, we anticipated that psilocybin enhances learning with neutral and conscious stimuli. Lastly, we speculated that due to a reduction in top-down processing with psychedelics,^39^ subconscious cues, which demand greater attention,^34^ disrupt learning to a higher degree with psilocybin than with placebo.

## Results

### Overall performance of psilocybin is noninferior to placebo

**Figure 1:**
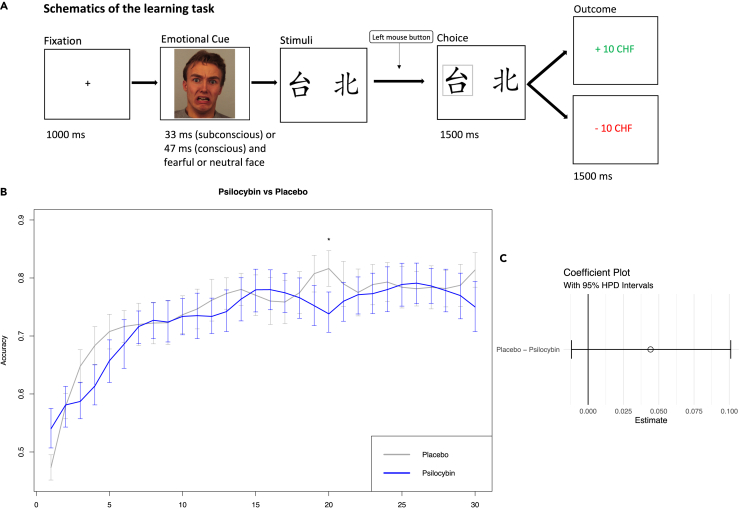
The learning rate of psilocybin is noninferior to placebo To introduce learning in uncertainty, the probabilistic component of the task returned a positive reward of 70% for the more lucrative symbol and a loss of 30% of the time, while for the less lucrative symbol, the percentage was reversed. Each subject solved eight tasks in total. Four on each measurement day (psilocybin or placebo). Each task consisted of sixty trials evenly distributed among two symbol pairs, leading to a total of 240 data points per trial and per condition. The conditions were a tupel of (subconscious, fearful), (subconscious, neutral), (conscious, fearful), and (conscious, neutral) (A). In the first trial, the more lucrative symbol was chosen with an accuracy of approximately 50%. Progressing through trials, the accuracy of choosing the “better” symbol increased logarithmically, reflecting a learning effect. Each learning curve was constructed from 30 subjects completing four tasks, each involving two symbol pairs, resulting in a total of *n* = 240 per trial and substance and *n* = 7200 per substance. (B) Overall learning curve with the logarithmic smoothing of placebo versus psilocybin. Psilocybin is shown in blue and placebo in gray. The mean on each trial is depicted with standard errors. Only trial number 20 showed a significantly better result in favor of placebo. (C) Psilocybin did not significantly differ from placebo. Coefficient plot of placebo vs. psilocybin reflecting a lack of overall significant difference (0 ∈ 95% HPD). Asterisks denote statistical significance (∗*p* < 0.05).

Our measure of learning consisted of the positive change in accuracy (from 0 to 1) when determining the more lucrative symbol out of two in a learning task (Figure 1A). We calculated the accuracy in each task for each substance and plotted its change as a learning curve (Figure 1B). At the beginning of each task, both symbols held equal uncertainty, leading the learning curve to commence at approximately 50% accuracy at trial 1 and progressing until trial 30, reflecting a learning effect. Trial-to-trial comparisons showed no significant difference between psilocybin and placebo, except trial number 20 favoring placebo (*p* < 0.005, Mann‒Whitney-test, corrected for multiple comparisons).

As a main result, the learning effect between psilocybin and placebo across all conditions did not reach significance (0 ∈ 95% HPD) (Figure 1C). Psilocybin led to a mean accuracy score over all trials of 0.729 (*N* = 30, *n* = 3600, σ = 0.332), while the overall placebo accuracy score was 0.745 (*N* = 30, *n* = 3600, σ = 0.324). The Hodges‒Lehmann estimator indicating the median of all pairwise differences was 0, while the effect size (Cliff’s delta) was 0.023, faintly favoring placebo. Moreover, in the Levene’s Test for Homogeneity of Variance, the variance of psilocybin showed borderline significance (*p* = 0.05002, *N* = 30, *n* = 7200) compared to placebo, suggesting a higher amount of switching and a greater flexibility in responses in the psilocybin group.

### Subconscious cues disrupt performance of psilocybin

**Figure 2:**
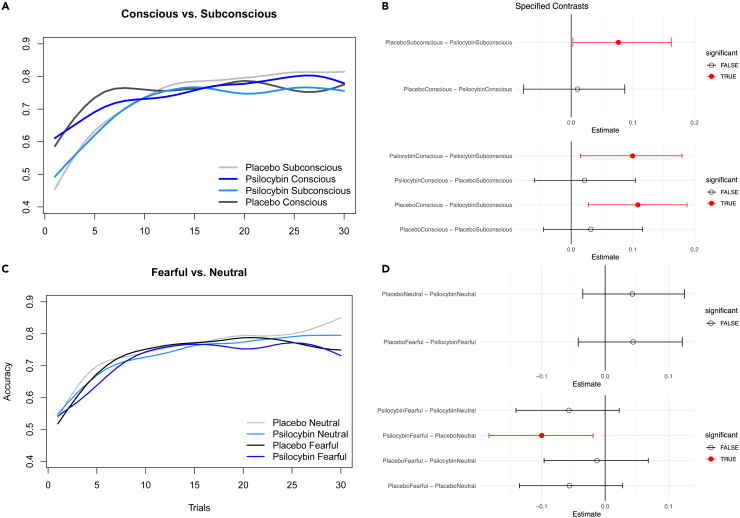
Learning curves of different conditions Learning curves of subdivided conditions (duration of presentation, emotional cues), comprising 30 subjects solving two tasks with two symbol pairs, leading to *n* = 120 per trial and condition to a total of *n* = 3600 per condition. Psilocybin is shown in shades of blue and placebo in shades of gray. (A and B) Learning curve of subconscious compared to conscious cues with a (B) coefficient plot of pairwise marginal mean differences of direct comparisons at the top, showing significance between subconscious cues in favor of placebo, and psilocybin conscious and placebo conscious over psilocybin subconscious (0 ∉ 95% HPD respectively). (C and D) Learning curves of neutral and fearful cues with a (D) coefficient plot of pairwise marginal mean differences of direct comparisons at the top, showing significance between placebo neutral over psilocybin fearful (0 ∉ 95% HPD respectively).

We calculated the pairwise marginal means from fitted models *of fearful* vs*. neutral* faces and *subconscious* vs. *conscious* presentation of cues. The mean accuracy score over all trials for subconscious cues with psilocybin was 0.714 (*N* = 30, *n* = 1800, σ = 0.337), whereas for placebo it was 0.739 (*N* = 30, *n* = 1800, σ = 0.327). The accuracy improvement of cues with different presentation times was plotted in a learning curve (Figure 2A). Regarding presentation times, psilocybin’s performance with subconscious cues was significantly worse not only against placebo but also against the other three conditions (0 ∉ 95% HPD for all three) (Figure 2B). Similarly, we plotted the learning curve of emotional cues. With placebo, fearful and neutral cues led to a similar learning curve up to trial number 22, where they start to diverge (Figure 2C). The mean accuracy score for psilocybin-neutral was 0.737 (*N* = 30, *n* = 1800, σ = 0.332), whereas for placebo-fearful it was 0.754 (*N* = 30, *n* = 1800, σ = 0.333). With emotional cues, psilocybin’s performance with fearful faces was not significantly different against placebo (0 ∈ 95% HPD), while being significantly worse to placebo-neutral (0 ∉ 95% HPD) (Figure 2D).

### Subconscious neutral cues hinder learning of psilocybin while conscious neutral cues facilitate learning in psilocybin

**Figure 3:**
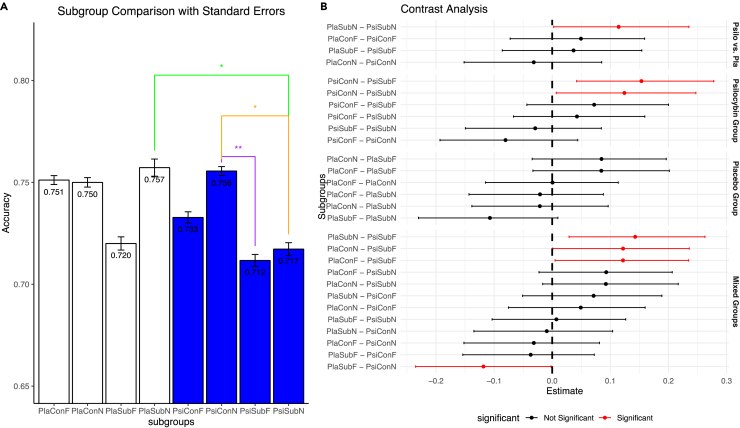
Subconscious fearful cues hinder learning in placebo and conscious neutral cues facilitate learning in psilocybin (A) Bar graphs of subgroups with standard errors (each with *N* = 30, *n* = 1800). Direct comparison led to subconscious neutral cues with psilocybin faring worse compared to placebo subconscious neutral (green, 0 ∉ 95% HPD), while group comparison with psilocybin led to conscious neutral cues performing better than both subconscious neutral (orange, 0 ∉ 95% HPD) and subconscious fearful (purple, 0 ∉ 99% HPD). (B) Coefficient plot of the pairwise marginal means of the performance comparison of direct subgroups, substance groups and mixed pairs, with significant differences highlighted in red. Legend: *Pla = placebo, Psi = psilocybin, Con = conscious (47 ms), Sub = subconscious (33 ms), F = fearful, N = neutral.* Asterisks denote statistical significance (∗0 ∉ 95% HPD, ∗∗0 ∉ 99% HPD).

When comparing the performance of psilocybin and placebo, subconscious neutral cues with psilocybin led to a mean accuracy of 0.717 (*N* = 30, *n* = 900, σ = 0.342), while for placebo 0.757 (*N* = 30, *n* = 900, σ = 0.327) with a statistically significant higher performance of placebo (0 ∉ 95% HPD). The other combinations of emotions and duration of presentation did not reach statistical significance (0 ∈ 95% HPD). Performance comparison among the psilocybin group with different cue-pairs led to conscious-neutral cues faring better than both subconscious-neutral and subconscious-fearful group (0 ∉ 95% HPD for both), while the placebo group did not show statistical difference (0 ∈ 95% HPD). Moreover, the comparison of mixed cues led to the following results: placebo-subconscious-fearful fared worse than psilocybin-conscious-neutral (0 ∉ 99% HPD). Psilocybin-subconscious-fearful was inferior to placebo-conscious-neutral, placebo-conscious-fearful, placebo-subconscious-neutral, and psilocybin-conscious-neutral (0 ∉ 95% HPD for all four) (Figures 3A and 3B).

### Receiving psilocybin first in the crossover task impeded overall performance

**Figure 4:**
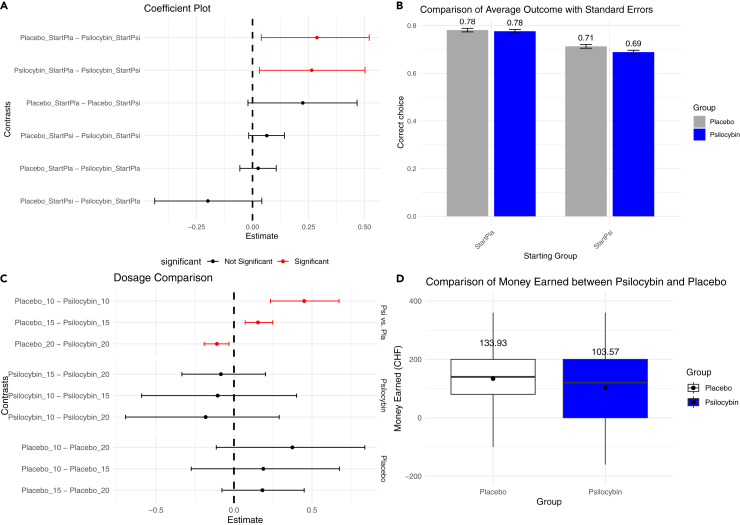
Placebo earned more money, and performed better when administered first, while higher dosage of psilocybin led to better results Receiving placebo first led to higher scores. The group receiving psilocybin first scored significantly worse than those receiving placebo first and those psilocybin after placebo (0 ∉ 95% HPD) (A) and bar graph reflecting the difference in scores (B). Higher dosage of psilocybin led to higher scores than the placebo. Coefficient plot of pairwise marginal means of different dosages (C). The 20 mg psilocybin group had significantly higher scores than the same participants receiving a placebo (0 ∉ 99% HPD). The 15 mg placebo group achieved a significant score over the 15 mg psilocybin group (0 ∉ 99%). Although only limited analysis is possible due to the small sample size (*N* = 2, *n* = 60), the 10 mg-placebo group was superior to the 10 mg psilocybin group. Placebo led to higher earnings than with psilocybin. Boxplot of average earnings per task of psilocybin vs. placebo (D). With psilocybin, participants earned almost one-third less on average than placebo, although this difference did not reach statistical significance (*p* = 0.09, Mann‒Whitney-test, *n* = 30, *N* = 7200 per substance). Moreover, the total amount of monetary gain was fraught with probabilistic undulations, since participants were also probabilistically able to earn money by choosing the inferior symbol. The horizontal bar shows the median, and the black dot shows the mean. Legend: *StartPla* denotes the group of participants receiving placebo first and *StartPsi* received psilocybin first.*15* = 15 mg, *20* = 20 mg.

Participants receiving psilocybin on the first day (μ = 0.689, *n* = 16, *N* = 3840) achieved significantly worse results than both those receiving psilocybin after placebo (μ = 0.776, *n* = 14, *N* = 3360) and placebo on the first day (μ = 0.781, *n* = 14, *N* = 3360) (0 ∉ 95% HPD for both) (Figures 4A and 4B).

### Higher dosage of psilocybin outperforms placebo

We compared the dose-dependent performance with a Bayesian mixed-effects regression model with a beta distribution. We split participants into three dosage levels and compared their scores with psilocybin and placebo. Although dosages were weight-adapted into three categories, the 20 mg psilocybin group (μ = 0.74, *N* = 15, *n* = 540) significantly outperformed the same group receiving placebo (μ = 0.725, *N* = 15, *n* = 540) (0 ∉ 99% HPD). The 15 mg psilocybin group (μ = 0.717, *N* = 13, *n* = 390) had the opposite result, with significantly inferior scores compared to the same group receiving placebo (μ = 0.747, *N* = 13, *n* = 390) (0 ∉ 99% HPD). While the 10 mg group counted only two participants (*N* = 2, *n* = 60) and the analysis of that dosage level is limited, the higher dosage of psilocybin fared better against their placebo counterpart (Figure 4C).

### Similar intrapersonal performance of placebo and psilocybin in the crossover task

Comparing the randomized crossover *intra*personal results of each participant’s performance against themselves of psilocybin vs. placebo, 12 participants (40%) achieved a significantly higher accuracy with placebo (*p* < 0.05, Mann‒Whitney test), while 7 participants (23.3%) performed significantly better with psilocybin (*p* < 0.05, Mann‒Whitney test), and 11 participants (36.7%) did not show a significant difference between the two substances (*p* > 0.05, Mann‒Whitney test).

### Higher nonsignificant monetary reward with placebo

The monetary reward between the psilocybin and placebo groups did not reach significance (*p* = 0.09, Mann‒Whitney test). The placebo group managed to earn 133.9 ± 9.9 CHF (mean ± standard error), while the psilocybin group reached 103.5 ± 12 CHF. Although the monetary gain was fraught with probabilistic undulations, with placebo participants gained 29.3% more money than with psilocybin (Figure 4D).

### Faster reaction times with a higher dose of psilocybin, while overall slower than placebo

Regarding the reaction times, on psilocybin, we observed a mean of 1.73 s per trial (σ = 2.11 s). On placebo, we observed a mean of 1.34 s (σ = 1.62 s). Placebo led to significantly faster reaction times (*p* < 0.001, Mann‒Whitney test). The Hodges‒Lehmann estimator found that on average, psilocybin led to 0.36 s longer reaction times. Interestingly, the reaction times of the 20-mg group were statistically faster than those of the 15-mg group (1.49 s vs. 2.01 s, *p* < 0.001, Mann‒Whitney test).

### Psilocybin led to higher subjective vigilance reduction and impaired control and cognition

In psychometric scales, the dimension Vigilance Reduction of the 5D-ASC scale placebo reached a mean score of 9.75 compared to 44 under psilocybin (*p* < 0.001, Mann‒Whitney-test), a Cohen’s d of 1.97, and a 0.32 Pearson correlation coefficient. In the 11D-ASC questionnaire, the dimension Impaired Control and Cognition, placebo had a mean score of 0.74 compared to 29.48 in psilocybin with a significant difference (*p* < 0.001, Mann‒Whitney-test), a Cohen’s d of 1.52, and a 0.1 Pearson correlation.

## Discussion

In this study, we investigated the effects of psilocybin on strategy finding in a probabilistic learning task next to the impact of various cues on awareness. We showed that psilocybin did not impede reinforcement learning by modulating serotonin signaling in the brain against placebo and that subconscious cues disturbed performance with psilocybin. As participants traversed trials, selecting the symbol associated with monetary gain resulted in an upward trajectory of the learning curve. Both the psilocybin and placebo groups exhibited a learning curve at a comparable rate, without reaching statistical difference. Of the thirty trials only one was significant in favor of placebo, which we attributed to random chance. Despite being a powerful hallucinogen that alters thought patterns and emotions, the capacity to navigate an uncertain environment and to update beliefs effectively prevailed in different tested dosages of psilocybin.

Emotional cues from fearful faces signal unconditioned threat-related stimuli and have led to a performance boost in other studies by serving as a conditioned predictive stimulus in cognitive processing.^40^ Contrary to previous findings, in our task, fearful faces did not contribute to an enhancement in the performance of placebo or psilocybin, and along neutral faces, exhibited similar learning rates when comparing placebo vs. psilocybin. In other studies, acute effects of psilocybin were found to diminish the perception of negative emotions due to reduced activity in right amygdala activity,^41^ while simultaneously correlating with increased positive mood,^42^ and leading to 5-HT–mediated DA enhancement, facilitating fear extinction learning.^43^ In depression, there is a bias of processing negative over positive stimuli by deficient 5-HT activity.^40^ Similarly, this reduced perception and weakened amygdala blood-oxygen-level-dependent (BOLD) response of fearful faces was observed with both acute^44^^,^^45^ and repeated SSRI administration.^46^^,^^47^ The weakened recognition of fearful faces was blocked by the 5-HT_2A/2C_-receptor antagonist ketanserin,^21^ indicating the role these receptors play in mediating these effects. An *increase* in 5-HT transmission (e.g., prolonged SSRI administration) results in attenuated aversive processing and a reduced BOLD response of the amygdala to fearful faces, while *reduced* 5-HT levels (e.g., acute tryptophan depletion, acute SSRI administration) augment aversive processing, heighten fear recognition, and diminish reward learning.^40^ Consequently, 5-HT depletion leads to heightened reactivity to aversive signals, prioritizing punishment over reward accompanied by increased impulsivity.^48^ While the acute effects on the amygdala of psilocybin resemble those of prolonged SSRI administration, the latter have been found to impair learning by blunting emotions,^36^^,^^37^ whereas psilocybin enhances the perception of positive emotions.^49^

Psilocybin resulted in 5% higher variance difference than placebo, which was borderline significant, suggesting a slightly higher amount of switching under psilocybin and pointing at a higher exploratory behavior. Similarly, LSD was shown to decrease stimulus stickiness in a learning task,^18^ hinting to an increased flexibility in decision-making of serotonergic psychedelics. This resembles acute single SSRI administration, which reduced 5-HT transmission in a probabilistic learning task and led to a higher tendency to inappropriately switch a strategy after punishment. Thus, in our study, we observed effects of psilocybin in line with both acute (inappropriate switching) and chronic (reduced aversive processing) SSRI administration, pointing at mixed effects possibly due to its partial agonism at different 5-HT receptors. Another role of serotonergic transmission in learning is that 5-HT is thought to interact with DA neurons by encoding the beneficialness^50^ of a given action or outcome through promoting goal-directed behavior, assessing the level of satiation, and preventing impulsive and appetitive actions while anticipating a reward. Thus, 5-HT may be indicative of temporal discounting^51^^,^^52^ and signal an individual’s motivation to either maintain or switch to a different behavior based on the perceived benefits of each option.^50^ Higher 5-HT levels led to less impulsive actions and increased acceptance of a delayed reward, compared to a bias toward smaller immediate rewards with lower 5-HT levels.^52^^,^^53^

Regarding the learning dynamics of varying presentation times, conscious cues corresponding to the cortical pathway notably engendered better results under psilocybin than cues presented subconsciously via the subcortical pathway. This result was reflected in other studies speculating that subconscious cues may evoke a feeling of uncertainty or anxiety,^54^ yielding less ideal choices. Subgroup analysis expanded on this result. Although subconscious cues overall hindered learning with psilocybin, it was only the neutral cues presented subconsciously under psilocybin — not subconscious fearful ones — that led to worse performance compared to placebo. Moreover, despite fearful and neutral cues equating performance in the psilocybin group, only conscious neutral cues were superior to both subconscious emotional modalities with psilocybin, pointing at a destabilizing performance of fearful faces with psilocybin in the conscious condition and a favorable in the subconscious condition. The remaining results, which compare mixed pairs (e.g., placebo subconscious neutral against psilocybin conscious fearful) are speculative, since comparison might not be straightforwardly applicable.

In informational processing, task-irrelevant visual information is initially processed in visual areas and subsequently suppressed through top-down attentional processing.^54^ Such a process demands more attention to accommodate subconscious cues accordingly.^54^ Reduced top-down processing under psilocybin^55^ might attenuate the suppression of task-irrelevant visual information, leading to a larger learning impairment of subconscious cues.^56^ 5-HT activity at the subcortical level could induce motivational processes opposed to those mediated by DA by increasing punishment PE. However, in our study, only participants under psilocybin exhibited a significantly superior learning effect with conscious cues compared to subconscious cues and were more adversely affected by subconscious cues than those receiving placebo, hinting at a possible serotonergic distinction in these pathways.

For example, the 5-HT_1A_-receptor is reputed to be involved in disturbing attentional tracking,^20^ while the 5-HT_1A_ and 5-HT_2A_-receptors exert opposite actions in the mPFC.^20^^,^^57^ These contrasting findings point at the multifaceted pharmacological actions of psilocybin leading to substantial alterations in awareness and self-related cognitive processes in cortical-subcortical functional networks, such as within the prefrontal cortex (PFC), posterior cingulate cortex (PCC) and reducing other regions across the default mode network (DMN).^58^ Moreover, the 5-HT_2A_ receptor is the main receptor activated by psilocybin and the predominant 5-HT subtype in the cortex and the DMN, especially in the PFC.

Predictive coding indicates that top-down signaling is linked with prior expectations of lower-level neural activities, while bottom-up signaling conveys PE.^59^^,^^60^ Psychedelics are believed to relax potentially pathologically overweighted priors by decreasing the top–down inhibitory control of the PFC and PCC,^39^ while simultaneously enhancing bottom-up information flow. These alterations correspondingly manifest a richer phenomenological experience and facilitate therapeutic insights by enabling new perspectives.^55^ A discrepancy of either top-down or bottom-up mediated signaling could result in aberrant behavior leading to learning impairments. Dysfunctional top-down signaling could distort sensory experience, up to possibly inducing hallucinations in the absence of a stimulus. Meanwhile, deviations in bottom-up signaling may indicate imprecise prior beliefs, requiring adaptation to accurately model the current experience of reality.^61^

The NMDA receptor antagonist ketamine was found to disrupt top-down control of prior expectation signaling in cortico-hippocampal circuits, leading to dysfunctional and overly precise bottom-up prediction error signaling, manifesting a failure in sensory attenuation and false inference cycles.^62^ In studies examining the effect of psychedelics on sensory stimuli, ketamine was shown to disrupt the generation of auditory event-related potential (ERP) mismatch negativity (MMN) and impair performance in a continuous performance task.^19^ The MMN reflects changes in PE and is a measure of learning.^63^ In the same task, psilocybin exhibited similar performance deficits while preserving MMN and lowering ERP in N100.^19^ Additionally, another study reported analogous disruptions in N100 and P300 due to psilocybin.^64^ This points to specific roles of the NMDA receptor in impairments of MMN^19^^,^^65^ and the 5-HT_2_ receptor in ERP alterations. The observed performance deficits in the continuous performance task for both substances were hypothesized to originate from shared downstream dopamine^5^ and excessive glutamate release.^19^ With tactile stimuli, psilocybin was found to weaken both the MMN response to surprising stimuli and the integration sensory inputs via aberrant PE processing, associated with alterations in subjective body and self-experience.^66^ Since the P300 amplitude correlates with learning,^67^ and psilocybin was found to dose-dependently reduce P300,^64^ it would be expected to see reduced learning effects with increasing dosages of psilocybin. However, we observed no significant reductions in learning against placebo, and notably, we even observed enhanced learning with a 20 mg dosage of psilocybin compared to placebo. In summary, psilocybin-induced 5-HT_2_-receptor signaling generally maintains MMN for most tested stimuli, except for a decrease observed with tactile inputs. This reflects a sufficiently functional learning system in line with our findings of preserved learning in a probabilistic learning task. Moreover, alterations in cortico-subcortical signaling might reflect our findings of altered learning effects of conscious and subconscious cues.

Similar to previous findings,^42^ psilocybin led to slower reaction times than placebo. This effect was more pronounced for negative and neutral stimuli but not for positive stimuli.^21^ Surprisingly, when comparing the learning rate of the 20 mg group of psilocybin not only did it perform significantly better than the placebo counterpart but also exhibited faster reaction times than the 15 mg group of psilocybin. In a previous study, LSD exhibited both an increased punishment and reward learning rate compared to placebo in a learning task.^18^ While we observed learning improvement only with 20 mg dosages of psilocybin against placebo, the lack of difference in other variables might be due to our task specificity, the broader pharmacological profile of LSD^1^ (additional action at dopaminergic and adrenergic receptors), or the disparity in the used model.

When considering the order of substance administration, starting the task with psilocybin yielded less favorable results compared to both commencing with placebo or receiving psilocybin in the second session. This could imply higher initial uncertainty with psilocybin that diminished as the task progressed and suggests the importance of starting an unfamiliar task in a sober state. Thus, participants who received placebo first might have benefited from an initial ordinary cognitive experience, better preparing them for the subsequent session with psilocybin. In contrast, participants who received psilocybin first had to adapt to performing the task while sober. The monetary reward did not reach a statistical difference between psilocybin and placebo. Despite used as a main incentive for determining the correct choice, the total sum of money earned in the task did not pose a reliable measure of learning due to participants being able to earn money with a 30% chance even when selecting the incorrect symbol, respectively facing a 30% chance of loss with the correct symbol. Another reason for the difference could be that some participants noted psilocybin diminished the allure for monetary gain as money lost its importance and the pleasure derived from monetary gain was diminished compared to an ordinary waking state. In a future study, a more suitable incentive for psilocybin might be used to assess learning effects.

Although the psychometric evaluation of the psilocybin group did report a subjective impairment of vigilance and cognition, their objective performance did not reflect this, as performance did significantly differ from placebo. This suggests that the underlying mechanism of decision-making and strategy finding sufficiently prevails in a probabilistic learning task, contrary to psychometric self-assessments. In contrast, an opposite pattern is seen with nootropics or neuroenhancers, where individuals report subjective cognitive improvement without objective enhancement.^68^

Of our hypotheses, we showed that first, fearful faces might impede learning with psilocybin only when consciously perceived. Second, conscious and not neutral stimuli led to better results with psilocybin. Lastly, subconscious cues led to a worse performance with psilocybin than with placebo, due to a reputed higher disruption of attention with psilocybin.

### Conclusions

The manifold potential applications for psilocybin in treating psychiatric conditions involves a complex interaction between neurobiological changes and psychological insights – among others through the interplay of 5HT_2A_-agonism,^24^ neuroplasticity,^16^^,^^17^ relaxed rigid beliefs,^39^ and decreased DMN activity^58^ – leading to a revision of maladaptive thought patterns. Our study was the first to test psilocybin in a probabilistic learning task and to explore some of these dynamics. We found that the awareness-altering effects of psilocybin with all tested dosages adequately preserved the capacity for strategy finding and decision-making against placebo, especially with a higher dosage. This suggests that learning in an uncertain environment under therapeutic sessions of psilocybin can sufficiently take place. Moreover, subconscious cues diminished learning with psilocybin, likely stemming from a hypothesized shift in top-down and bottom-up mediated signaling with psychedelics. Further research is needed to better understand the mechanisms underlying therapeutic learning effects and to determine the optimal dosing and administration protocols for psilocybin and other psychedelics for this purpose.

### Limitations of the study

The limitations of the study were first, the complexity of the design and the amount of data measuring different hierarchical subgroups did not allow for the implementation of a proper reinforcement learning model, leading to a Bayesian mixed-effects regression model instead. Second, performance was measured approximately three to 4 h post-substance intake, where acute physiological psychometric changes only prevail at approximately 50%.^69^^,^^70^ Third, the effects of psilocybin contributing to cognitive and neural flexibility in patients suffering from depression revealed a surprising lack of correlation between the improvement of depression and enhancement of cognitive capabilities.^71^ It is speculated that heightened flexibility, a major part of the therapeutic effects of psilocybin, creates a window of plasticity facilitating improvements, akin to escaping a dysfunctional rigid state (by flattening local minima as suggested in the REBUS model^39^). This aspect is potentially not fully captured in a probabilistic learning task.

## STAR★Methods

### Key resources table

**Table:** 

REAGENT or RESOURCE	SOURCE	IDENTIFIER
**Software and algorithms**		

MATLAB 2021a	https://ch.mathworks.com/products/matlab.html	RRID:SCR_001622
Presentation®	https://www.neurobs.com/	N/A

### Resource availability

#### Lead contact

Further information and requests for resources should be directed to and will be fulfilled by the Lead Contact, Andrea Casanova (andrea.casanova@bli.uzh.ch).

#### Materials availability

This study did not generate new unique reagents.

#### Data and code availability

• Data reported in this paper may be shared by the lead contact upon request.
• Original code was written for EmotLearn in Presentation® and may be shared by the lead contact upon request.
• Any additional information required to reanalyze the data reported in this paper is available from the lead contact upon request.

### Experimental model and study participant details

The experiment constituted a randomized, double-blind crossover placebo-controlled study using a low (10 mg; <50 kg body weight) mid (15 mg; <80 kg) or a high dose (20 mg; ≥80 kg) of psilocybin tested against a placebo (mannitol). A total of 30 white healthy right-handed (23 male and 7 female) volunteers with a mean age of 29 years were enrolled. Exclusion criteria were personal and family history of major psychiatric disease as defined in the DSM-V, any major medical conditions, family history of seizure disorder, current psychopharmacological treatment or use of medication, and pregnant or breastfeeding women. The data were gathered as part of the study *Characterization of Altered Waking States of Consciousness in Healthy Humans (NCT03853577)* at the Psychiatric University Hospital Zurich between 2019 and 2021. All participants provided written informed consent. The experimental protocol was approved by the local ethics committee of Zurich (BASEC Nr.: 2018-01866). The use of psilocybin was authorized by the Swiss Federal Office of Public Health.

### Method details

#### Procedures

As part of the main study, participants were investigated double-blind and randomized twice at a 14-day interval with TMS-EEG after administration of psilocybin or placebo (mannitol) and approximately 240 min after substance intake solved a probabilistic learning task, EmotLearn, where the goal was to maximize virtual monetary reward while different emotional cues were presented. Participants were instructed on the specific process and goals of the learning task both on the screening day and on the test day before starting the task. Because of time restraints, no test session was held during the testing day. To counter order effects and minimize systematic bias, we applied complete and random counterbalancing by both randomizing the order of tests on a given day and randomly assigning a participant to one of the two substances.

#### EmotLearn

EmotLearn is a probabilistic learning task programmed in Presentation (https://www.neurobs.com/) to examine the computational processes behind the interaction between reward learning and subconscious/conscious emotional processing. The goal was to estimate how different emotional cues affect the learning rate under psilocybin or placebo. These emotional stimuli were female and male fearful and neutral faces taken from the Karolinska Directed Emotional Faces database (https://www.kdef.se/), presented in a random order. On any of the two measurement days, each participant solved four tasks consisting of sixty trials. On each task, a fixed condition pair was tested (subconscious-fearful, subconscious-neutral, conscious-fearful, conscious-neutral). Overall, the thirty participants accumulated 7200 data points per substance (psilocybin, placebo). Each task consisted of an emotional cue followed by one out of two symbol pairs, presented thirty times each. The design was inspired by a study categorizing different presentation times into subconscious (33 ms) or conscious (47 ms) perception,^32^ reflecting two hypothesized pathways of emotional processing.^56^ A slower, more precise, cortical pathway (several visual stages such as the retina, lateral geniculate nucleus of the thalamus, primary visual area cortex, higher-order brain areas, and finally extending to the amygdala), and a faster, coarser, subcortical pathway (leading through the retina, superior colliculus, pulvinar nucleus of the thalamus, and extending to the amygdala).^56^ Each trial began with a fixation cross (1000 ms), followed by an emotional cue in the form of a face. Participants chose one of two symbols by pressing the left or right mouse button to progress to the next trial. Their choice was highlighted with a gray square around the symbol (1500 ms) followed by the outcome (1500 ms) in the form of +10 CHF reward or −10 CHF loss. Each symbol pair was endowed with a superior and an inferior symbol. The superior symbol had a higher probability (70%) of returning a positive reward (+10 CHF) and a lower probability (30%) of returning a loss (−10 CHF), while the opposite was true for the inferior symbol (70% for 10 CHF loss and 30% for 10 CHF reward). A task was concluded when a subject completed sixty choices (two symbol pairs presented thirty times each). Each symbol was randomly displayed an equal number of times on the left or right, with sixteen different symbol pairs randomly assigned to a task. Participants had to learn the stimulus-outcome associations and find the most lucrative strategy to maximize their virtual money reward, although no money was paid out and served as an indirect incentive. Participants usually determined their own learning heuristic as a win-stay, lose-switch strategy often applied in learning tasks.^31^ This probabilistic flair needed the participants to quantify uncertainty and flexibly update their beliefs in an environment of incomplete data.

### Quantification and statistical analysis

Raw data from the logfiles consisting of ratios of correct choices between 0 and 1 reflecting the accuracy of choosing the more favorable symbol on each trial in the learning task were extracted with *Microsoft Excel* (Microsoft, version 16.44) and *MATLAB* (MathWorks, version 6.5). The Shapiro‒Wilk test determined that the data from the learning task were not normally distributed (*p* < 0.05). Learning curves of accuracy of correct choices were smoothed with a Gaussian filter. All analyses were performed with *R* (R Core Team, version 4.3.1). For psychometric analysis of learning performance, we used the main dimension “Vigilance Reduction” of the 5-dimensional Altered States of Consciousness Questionnaire (5D-ASC)^72^^,^^73^ and the subdimension “Impaired Control and Cognition” of the 11 s-order scales of the 5D-ASC. The nonparametric scores were calculated with a Mann‒Whitney test comparing scores after the psilocybin and placebo sessions. The significance level used α = 0.05 in the null hypothesis.

#### Modeling and computational analysis

We employed a Bayesian mixed-effects regression model with both fixed and random effects considering an interaction term and applied a prior distribution. Since our main accuracy measure was the proportion of correct choices bounded between 0 and 1, we used a beta distribution for the dependent variable. For each group hierarchy (placebo vs. psilocybin, cue category (fearful vs. neutral, subconscious vs. conscious, dosages) and subgroup (e.g., placebo conscious neutral vs. psilocybin subconscious fearful)), we fitted multiple models in *R* using a hierarchical Bayesian method with Hamiltonian Markov chain Monte Carlo sampling using the No-U-Turn Sampler and implemented in *Stan* (Version 2.17.3). The model was run with four chains, each with 2000 iterations, and the first 1000 iterations of each chain were discarded as warm-up, leaving a total of 4000 post-warmup draws for inference. Models were compared with different priors, and the mean of the prior was calculated with the logit function. Posterior distributions were interpreted using the 95% Bayesian credible interval called the highest posterior density interval (HPD). All models attained a value of $\overset{ˇ}{R}$ = 1.0, indicating convergence. Model comparison was performed with Akaike-Information-Criteria (AIC) and Watanabe-AIC (WAIC) after they were fitted with different fixed effects. The highest-level response variable consisting of the accuracy of correct choices denoted as ‘Outcome’ was modeled with a beta distribution with the mean μ linked to the predictors with the *logit* link function:

Outcome∼Beta(μ,φ)

$$logit\left(\mu \right)=\beta_{0}+\beta_{1}*DesiredGroup+\beta_{2}*Trial+\beta_{3}*Age+\beta_{4}*Dosage+\beta_{5}*Starting+\beta_{6}*\left(DesiredGroup*Trial\right)+b_{i}$$

$\beta_{0}-\beta_{6}$ are fixed effect coefficients, b_i_ represents the random effects for the individual i. The constant precision parameter φ was modeled with an identity link. Predictors at the observation level were in the form of fixed effects, e.g., ‘Group’, ‘Trial’ (from 1 to 30 in a task), ‘Dosage’, ‘Gender’, ‘Age’, ‘Starting’ (which substance was first administered in the cross-over task) and random effects ‘(1|Individual i)’, capturing the between-individual variability in the outcome. For example, if the ‘DesiredGroup’ is the comparison of Dosages (‘GroupDosages’), the fitted model would look like this:

Outcome∼GroupDosages∗Trial+Age+Dosage+Starting+(1|Individuali).
